# Epigenetics alternation in lung fibrosis and lung cancer

**DOI:** 10.3389/fcell.2022.1060201

**Published:** 2022-11-07

**Authors:** Xueren Li, Chunjing Feng, Shouchun Peng

**Affiliations:** ^1^ Department of Respiratory Medicine, Tianjin Haihe Hospital, Tianjin, China; ^2^ Tianjin Institute of Respiratory Diseases, Tianjin, China; ^3^ The Institute Includes H&B(Tianjin) Stem Cell Research Institute, Tianjin, China

**Keywords:** epigenetics alternation, DNA, histone, lung, cancer, fibrosis

## Abstract

Respiratory disease including interstitial lung diseases (ILDs) and lung cancer is a group of devastating diseases that linked with increased morbidity and healthcare burden. However, respiratory diseases cannot be fully explained by the alternation of genetic information. Genetic studies described that epigenetic mechanisms also participate to transmit genetic information. Recently, many studies demonstrated the role of altered epigenetic modification in the pathogenesis of lung cancer and pulmonary fibrosis. Due to lacking effective medication, the underlying pathophysiological processes and causal relationships of lung diseases with epigenetic mechanisms still need to be better understood. Our present review provided a systematic revision of current knowledge concerning diverse epigenetic aberrations in major lung diseases, with special emphasis on DNA methylation, histone modifications, lncRNAs profiles, telomere patterns, as well as chromatin-remodelling complexes. We believed that a new target therapy for lung disease based on findings of the involved epigenetic pathway is a promising future direction.

## 1 Introduction

Respiratory diseases are the most common disease and public health problems in the world, especially after the COVID-19 outbreak in 2019. With increased air pollution, a large number of smokers, and an ageing population, it is a huge challenge that the prevention and treatment of respiratory diseases. At present lung cancer has become the most common malignant tumour in China. Another disease, pulmonary fibrosis is a risk factor for lung cancer. It is also confirmed with some common Pathogenic Mechanisms, has been gradually recognized and is the focus of current research (Tzouvelekis et al., 2019).

Lung cancer, a malignant tumour originating in the bronchial mucosa or glands of the lung, has significantly increased and remains the leading cause of death by cancer disease worldwide in recent years (McGuire, 2016). Traditionally, lung cancer can be classified into small cell lung carcinomas (SCLC) and non-small cell lung carcinomas (NSCLC) by histopathology (Herbst et al., 2008). Data from 339 cancer registries, the age-standardized incidence rate of lung cancer was 36.71 per 100,000 (Kobayashi et al., 2020). Epidemiology in China from 2012 to 2015 showed that the lung cancer survival rate in men and women was 16.8% and 25.1% respectively (Kropski et al., 2013). Compared to most countries, the mortality of lung cancer in China is relatively high because of tobacco abuse and air pollution. It may increase by approximately 40% between 2015 and 2030 in China (Martin-Sanchez et al., 2018). According to different pathological types and stages, the treatment strategy of each lung cancer is varied.

Pulmonary fibrosis (PF) is another fatal lung disease with an unknown pathogenic mechanism. PF eventually leads to respiratory failure and death of patients due to a progressive decline in pulmonary function (Tzouvelekis et al., 2019). Although the pathogenic mechanisms of PF are exclusive, repeated damage to AT1 cells and AT2 cells is a major component of PF. After lung injury, AT2 proliferate and then differentiate into AT1to repair normal alveolar structure and function in a normal physiologic reaction. Pre-alveolar type-1 transitional cell state (PATS), a transitional state that traverses between AT2 and AT1 was found in a recent study (Kobayashi et al., 2020). PATS undergo extensive stretching during differentiation, making them vulnerable to DNA damage, which is a feature associated with many chronic lung diseases, especially pulmonary fibrosis (Kropski et al., 2013). Idiopathic pulmonary fibrosis (IPF) is one of the most common ILD and its incidence is rising, with associated high morbidity, mortality, and limited therapeutic drugs (Barratt et al., 2018). IPF is a progressive scarring disease that resulted from ineffectual regeneration of the injured alveolar epithelial with abnormally elevated expression levels of transforming growth factor-β (TGF-β) signalling (Blackwell et al., 2014). It showed two discrete transitional states of AEC2-to-AEC1 differentiation after lung injury induced by LPS. The early stage is characterized by moderate and high expression of AEC1 markers, and the late is downregulation of AEC2 markers (Jiang et al., 2020; Riemondy et al., 2019).

Epigenetics, a term proposed by Conrad Waddington in the 1940s, was related to the concept of genomics. During the first three decades of its existence, it was little used in the sciences (Jablonka and Lamb, 2002; Waddington, 2012). By the end of the 20th century, epigenetics had rapidly developed and become a generally recognized branch of biology. Epigenetics deals with the heritable changes in gene regulation that is not related to the changes in the DNA sequence itself. A series of complex molecular mechanisms that mediate epigenetic phenomena, like DNA methylation, histone modifications, and Long non-coding RNA (LncRNA) are well-known processes.

In this review, we summarize current and emerging knowledge of epigenetics alternation in lung diseases, including fibrosis and cancer, and the potential future therapeutics for fatal lung diseases.

## 2 DNA methylation

DNA methylation is one of the best characterized epigenetic mechanisms. Specifically, the addition of a methyl group to the fifth carbon site of the cytosine base forms 5-methylcytosine (5 mC) (Yang et al., 2015). 5mC in the genome regulates many biological functions through gene expression regulation. DNA methylation dysfunction is closely related to the progression of autoimmune diseases, fibrosis diseases, tumours and cardiovascular diseases (Bakusic et al., 2017). It has been reported that DNA methylation plays an important role in the pathogenesis of pulmonary fibrosis and lung cancer. Thy-1 is an important factor in maintaining cell-stromal balance in normal lung fibroblasts, while Thy-1 expression is absent in IPF fibroblasts (Rege and Hagood, 2006). It has been reported that DNA methylation occurs in the promoter of Thy-1 during the pathogenesis of IPF to suppress the Thy-1 gene and enhance the anti-apoptotic ability of lung fibroblasts, resulting in Extracellular matrix (ECM) deposition and lung scar formation (Sanders et al., 2008). Pulmonary origin fibroblasts are characterized by a high level of Foxl1 expression. As a transcription factor, the Foxl1 gene in lung fibroblasts shows DNA hypomethylation and super-enhancer formation (Miyashita et al., 2020).

CpG islands (CGI) are CG-rich DNA segments, which are often located at or near the genes transcription start site (TSS). Typically CGI methylation state can regulate gene expression. DNA of tumour cells is globally hypomethylated, leading to activation of proto-oncogenes and increased genome instability (Salhia et al., 2010). Genome-wide DNA hypomethylation including CGI in cancer cells can activate the expression of proto-oncogenes that were previously silenced in normal tissues, while in which DNA hypermethylation inhibits DNA transcription, resulting in no or low expression of classic tumour suppressor genes (Salhia et al., 2010). Testing for hypermethylation in these genes could help early diagnosis of lung cancer. Many tumour suppressor genes and tumour-related genes have been reported to contain a variety of DNA methylation levels in lung cancer tissues, The methylation chip can integrate all known methylation sites on a single chip. The changes of methylation in the whole tumour and biomarkers in lung cancer were screened out and helped to better understand DNA methylation patterns during lung oncogenesis (Heller et al., 2010). Circulating cell-free DNA (cfDNA) extracted from small cell lung cancer can be used as a tumour surrogate to detect tumour genomic alteration (Mohan et al., 2020). Remarkably, cfDNA methylation profiling also has potential clinical utility for stage I–IV detection, disease monitoring and subtyping SCLC patients (Chemi et al., 2022).

In addition, DNA methylation alteration was also reported in other common respiratory diseases. Overexpression of NOS1AP and BID genes in lung tissues of smokers and COPD is strongly associated with altered DNA methylation status, which can disrupt cell senescence, autophagy, and apoptosis (Sundar et al., 2017). Hypermethylation of the SULF2 promoter region in sputum samples of smokers was associated with persistent high mucus secretion (Bruse et al., 2014). Regulatory T (Treg) cells are important immune response suppressors in the pathogenesis of asthma, which activity is regulated by a key transcription factor, forkhead box 3 (Foxp3). Increased DNA methylation of Foxp3 can seriously damage Treg cells (Nadeau et al., 2010).ORF8, similar motifs in the N-terminal tail region of ORF8 and histone H3, was the key factors that affect the modification of H3K9me3 and H3K27me3 and H3K9ac in host ells (Kee et al., 2022). ORF8 is highly expressed during infection, and interaction proteomic studies have found that ORF8 interacts with DNA methyltransferase DNMT1 in SARS-CoV-2 infection patients. (Kee et al., 2022; Thomann and Thiel, 2022).

## 3 Histone modification

Histones are the main structural proteins which can be wrapped by DNA to form a nucleosome. This close connection with DNA is efficient to package the genome into the nucleus, maintain genomic stability, and regulate gene expression through histone post-translational modifications (PTM) or histone variants dynamic. Therefore, regulation of gene expression through histone PTM is another important aspect of the epigenetics mechanism. Depending on the different modifications of terminal amino acids, histone modifications consist of acetylation, methylation, phosphorylation, and ubiquitination.

### 3.1 Histone acetylation and deacetylation

Histone acetylation is a typical epigenetic regulator for permissive gene transcription. Histone acetyltransferases (HATs) and histone deacetylation enzymes (HDACs) are the best-elucidated histone acetylation writers and erasers which regulate chromatin acetylation levels. Mounting evidence indicates that HDACs are involved in the pathogenesis of pulmonary fibrosis. Thy-1 is a cell outer membrane glycoprotein which exists in normal lung fibroblasts, but not in IPF fibroblast lesions (Rege and Hagood, 2006). In lung fibroblasts, repression of Thy-1 is both controlled by DNA hypermethylation and lower histone H4 acetylation levels in the Thy-1 promoter region (Korfei et al., 2015; Sanders et al., 2011). Immunohistochemistry showed that expression of almost all of HDACs was strongly induced in myofibroblasts of fibroblast and abnormal bronchiolar basal cells at the site of abnormal re-epithelialization of IPF, but not in control normal lungs (Korfei et al., 2015; Lyu et al., 2019).

HDAC4, an important HDAC in lung fibrosis tissue, can induce myofibroblasts to produce ECM. TGF-β1 could stimulate the expression of A-SMA in normal human lung fibroblasts while the HDAC4 level was attenuated by knocking down (Guo et al., 2009). To target dysregulated HDACs in pulmonary fibrosis, HDACs inhibitors were tested for their role in blocking the fibrosis process (Davies et al., 2012; Huang et al., 2013; Sanders et al., 2011). Inhibition of HDAC *in vitro* and *in vivo* models of pulmonary fibrosis has shown promising results, but advances in identifying pathogenesis and treatment strategies remain unclear.

Several lines of evidence also suggest that aberrant histone acetylation is likely to have a role in the EMT process of lung cancer. One group focuses on interactions of HATs/HDACs with EMT transcription factors and their effects on interstitial processes, such as p300, and PCAF. As one of the important HATs in cells, p300 is involved in the development of various tumours, and its mutations or disorders lead to a variety of diseases. The expression of many oncogeneses is also regulated by p300 (Pena et al., 2006; Yokomizo et al., 2011). p300/CBP-binding protein-associated factor (PCAF) also has the activity of histone acetylase and has the function of a transcription coactivator (Shiota et al., 2010). Although it remains to be determined whether alteration of HDACs is causal in EMT and tumorigenesis, nevertheless the expression level of HDACs in lung cancer and tumour EMT is different from that in normal tissues (Han et al., 2012). It can interact with EMT transcription factors and directly affect the expression of target genes to repress epithelial genes, leading to the occurrence of a mesenchymal process (Mamdani and Jalal, 2020; Tang et al., 2012). Therefore, HDAC inhibitors were also found can be used to inhibit EMT function and has a promising application prospect in the clinical treatment of lung cancer.

### 3.3 Histone methylation

All histone basic residues including arginines, lysines and histidines can be methylated. Histone methylation is an important part of epigenetics and leads to broad biological outcomes. In mammalian cells, histone methylation is known to have important roles in many biological processes, including transcriptional inhibition, transcriptional activation, DNA damage, and transcriptional extension. Catalyzed by histone methyltransferase (HMT), adenosine methionine methyl groups are transferred to histone H3 and H4 and covalently bound to lysine or arginine residues at the corresponding sites, resulting in chromosomal conformation changes (Kornberg, 1974), thereby diversifying gene expression. Among the Histone lysine methyltransferase (HKMTs) discovered so far, except HKMT4, all have a conservative SET domain. The most extensively studied histone methylation sites include H3K4, H3K9, H3K27, H3K36, H3K79 and H4K20 (Li et al., 2007).

In recent years, more studies found that abnormal histone methylation events caused by HMT play a causal part in tissue fibrosis. These findings provided a potential target for anti-fibrosis treatment. The occurrence of idiopathic pulmonary fibrosis is related to the deficiency of prostaglandin E2 (PGE2) and inducible COX-2 (Coward et al., 2014). Results showed that G9a-mediated H3K9 and EZH2-mediated H3K27 methylation levels were significantly increased in the COX-2 promoter region, resulting in COX-2 gene silencing in lung fibrocytes. Small interfering RNA (siRNA) of G9a and EZH2 can reverse their repressive epigenetic modification and restore producing PGE2 and COX-2 (Coward et al., 2014). These results reveal the role of G9a and EZH2 co-mediated histone modification in COX-2 apparent silencing, which is beneficial to understanding the pathogenesis of idiopathic pulmonary fibrosis. Methylation on Arginine site modification is also involved in the regulation of the fibrosis process. Protein arginine methyltransferases (PRMTs) proteins have been discovered, and PRMT1 is responsible for approximately 85% of intracellular arginine methylation (Campbell et al., 1996). It was confirmed that PDGF-BB can stimulate PRMT1 expression by enhancing the phosphorylation of ERK and STAT1. On the opposite, inhibition of PRMT1 activity can be achieved by AMI-1 down-regulates collagen fibre deposition and COX2 expression activated by the ERK pathway (Sun et al., 2016). This demonstrates the apparent regulatory role of arginine methylation in pulmonary fibrosis.

In general, the research on histone methylation of lung cancer mainly focuses on understanding the molecular mechanism. Methylation of lysine (K) and arginine (R) residues on histone tails largely determines chromatin configuration and biological outcome (Audia and Campbell, 2016). Methyltransferase ‘writers’ (KMTs) and the associated demethylase ‘erasers’ (KDMs) for histone lysine residue are termed histone lysine methyltransferases/demethylases. KMTs can remove methyl groups from lysine residues of histone or non-histone substrates (Moore and Gozani, 2014; Rea et al., 2000). Since the first histone KMT identified in humans is the H3K9 methyltransferase SUV39H1 (Rea et al., 2000), more KMTs have been discovered. Their methyltransferases are found to be closely associated with variant lung cancers except for essential roles in physiologic activities (Chen et al., 2018). SET Domain-Containing KMTs are closely related to tumorigenesis and disease progression in the lung. EZH2, the human homology of Drosophila, is the key catalytic component of the Poly-comb repressive complex 2. High levels of EZH2 and associated H3K27me3 are strongly related to poor clinical prognosis (Sato et al., 2013; Varambally et al., 2002; Wan et al., 2013). G9a is a KMT responsible for the mono- and di-methylation of H3K9 (Xue et al., 2018) and is highly expressed in invasive lung cancer cells. Expression of G9a can induce lung cancer progression in mice (Pandey et al., 2014; Xue et al., 2018). DOT1L, a Non-SET-Domain-Containing KMT, is the only known H3K79 methyltransferase and has structural similarities with PRMT1. H3K79 methylation was up-regulated in lung cancer cell lines, but the role of DOT1L in lung cancer is unclear (Rau et al., 2016). Until the finding of the first histone demethylase in 2004, Methylation of lysine or arginine residues was not regarded as reversible PTM (Shi et al., 2004). So far, more than 20 KDMs have been discovered and characterized, and many of them have been reported to be dysregulated in lung cancer. LSD1 (or KDM1A), the earliest reported and most studied KDM demethylase, exhibits abnormal overexpression and is a typical oncogene in lung cancer (Hayami et al., 2011). KDM2, a catalyzes demethylation on H3K36, was found highly dysregulated in 54 NSCLC cell lines and its mRNA and protein levels are significantly higher in primary NSCLC tumour samples than in the normal control (Wagner et al., 2013). Other KDM subfamilies associated with tumorigenesis in the lung, including KDM3A, KDM4A, KDM4D, KDM5A, and KDM6A (Sterling et al., 2020).

### 3.4 Histone ubiquitination

In 1975, Histone H2A was first found to be ubiquitinated at the highly conserved lysine residue 119 (K119) sites (Peng W. X et al., 2017). Ubiquitination of H2A promotes the binding of histone H1 to the nucleosome (Barratt et al., 2018), and plays an important role in the initiation of X chromosome inactivation (Goldknopf et al., 1975; Vijay-Kumar et al., 1987). Besides H2A, histone H2B can also be modified by ubiquitination. Ubiquitination of histone H2B promotes gene transcription (Wang H et al., 2004). Ubiquitin is a highly conserved 76 amino acid protein with a molecular weight of 8.5 kDa and is widely found in eukaryotes (Naito et al., 2009). Ubiquitination is a cascade of enzyme reactions catalyzed by ubiquitin activase (E1), ubiquitin-binding enzyme (E2), and ubiquitin ligase (E3) (Popovic et al., 2014).Ubiquitin E3 ligase and DUBs regulate pulmonary fibrosis by regulating TGF-β-dependent and independent pathways (Jaitovich et al., 2008; Li et al., 2018). Smurf 2, a type of E3 ubiquitin ligase, can can ubiquitinate and degrade its substrates and receptors, and then regulate TGF-β1/Smad signaling pathway in fibrosis (Chong et al., 2006). (Imamura et al., 2013).H2Bub1,a single ubiquitin added in the post-translational modification of histone H2B at lysine 120, have tumor suppressive effect through interection with cancer-related proteins.E3 complex RNF20/RNF40 is the main writer enzyme complex responsible for catalysing H2Bub1. H2Bub1 Loss was confirmed in some lung cancer (Urasaki et al., 2012; Zhang et al., 2017). Ubiquitination may also regulate the inflammatory responses initiating acute lung injury. E3 ubiquitin ligase plays an important role in inflammation and autoimmunity and has been shown to modulate acute lung inflammation caused by Toll-like receptor 4-mediated multifactorial sepsis (Chen et al., 2011; Zou et al., 2011).

## 4 Non-coding RNA

LncRNA has widely defined as non-coding RNA over 200 nucleotides in length, which are involved in the occurrence and development of many diseases. LncRNA regulates gene expression through a wide range of functions, including direct transcription, regulation of chromatin modification complexes, post-transcriptional regulation through processing, and acts like a molecular sponge leading to de-repression of miRNA target genes (Kung et al., 2013). LncRNA is associated with various organ fibrosis and has become a research hotspot to explore the role of lncRNA in the occurrence or progression of pulmonary fibrosis in recent years. It is believed that dual regulation of lncRNA in pulmonary fibrosis. The study demonstrated the potential role of lncRNAPCAT29 in the progression of pulmonary fibrosis. While lncRNAPCAT29 exerted key functions in silica-induced pulmonary fibrosis *via* the miR-221-TGF-β1-regulated RASAL1/ERK1/2 signal pathway (Liu et al., 2018). LncRNA is more involved in promoting the regulation of pulmonary fibrosis. Mir-26a was found to reduce TGF-β 1-induced upregulation of connective tissue growth factor (CTGF) in MRC-5 cells for inhibiting fibrosis (Liang et al., 2014). lncITPF expression is significantly upregulated in a transforming growth factor-β(TGF-β)1-smad2/3-dependent manner. It induces epigenetic regulation of host gene integrin β-like 1 by directly binding to heterogeneous nuclear ribonucleoprotein L. Clinical analysis showed that lncITPF was associated with clinicopathological features of IPF patients (Song et al., 2019). Telomere containing repeat RNA (TERRA) is a telomere containing lncRNA that is important for telomere stability. TERRA binds to the Shelterin complex in untransformed cells and regulates telomerase replication activity (Bettin et al., 2019). TERRA expression was increased in AT2 in ILD patients and bleomycin-induced fibrosis mice, and inactivation of TERRA improved cellular response in bleomycin-induced fibrosis mice (Gao et al., 2017). In summary, LncRNA plays a key role in mediating gene expression and cell function changes in the process of pulmonary fibrosis, both inhibiting and promoting the progression of pulmonary fibrosis, and the promoting function seems to be more significant.

At present, the pathogenesis of lung cancer has not been clear. Some scholars believe that lncRNA is involved in the occurrence and development of cancer, which may be a potentially important target for cancer diagnosis and treatment (Chi et al., 2019). lncRNA may prevent the interaction between miRNA and its downstream targets by binding corresponding miRNA, thus regulating the growth and metastasis of cancer, and lncRNA may serve as an important marker or therapeutic target of cancer (Peng W. X et al., 2017). At the same time, different types of lncRNA play different roles in lung cancer, which can be used as an important indicator to predict the susceptibility risk of lung cancer and for the diagnosis and treatment of lung cancer (Gong et al., 2016). For example, lung adenocarcinoma metastasis-associated transcription factor 1 is one of the first cancer-associated LNERNas identified and was initially considered a marker of survival in NSCLC patients (Ji et al., 2003). A study on the correlation between OCT4 and lncRNAs expression in 124 lung cancer patients found that overexpression of MALAT1 in lung cancer cells promoted cell proliferation (Jen et al., 2017). In addition, knockout MALAT1 inhibited OCT4-mediated lung cancer cell growth (Chiou et al., 2010; Jen et al., 2017).

## 5 Aging and telomere

Telomeres are important structural components of eukaryotic heterochromatin, which are located at the end of linear chromatin to resist DNA damage and prevent nuclear degradation (Riethman, 2008). In vertebrates, telomeric DNA has consisted of canonical TTAGGG repeats and their length wears away with cell division, limiting the self-renewal ability of the lung, and gradually leading to ageing, death, and apoptosis of alveolar cells (Alder et al., 2008). Recent studies have found that several lung diseases were causally associated with telomere dysfunction and cell senescence-related ageing. Telomere length and genomic integrity can be maintained by reverse transcriptase telomerase. Telomerase is a ribonucleic protein complex, which is mainly composed of three parts: telomerase RNA component (TR), telomerase reverse transcriptase (TERT) and telomerase-associated protein (TEP) (Alder et al., 2008; Borie et al., 2016). Much research has been focused on revealing the relationship between telomerase and the pathogenesis of IPF recent. Currently, TERT and TERC gene mutations are considered susceptibility markers of fibrotic lung disease (Armanios et al., 2007).73 patients with TERT and TR mutations from the Vanderbilt Familial Pulmonary fibrosis Registry were screened, and 6 patients had TERT or TR heterozygous mutations (Petrovski et al., 2017), TERT mutations were also found in 5% of sporadic IPF case studies. Studies on congenital dyskeratosis have proved that gene mutations of telomerase component factors can accelerate ageing, cause multi-system genetic diseases, and lead to premature death due to bone marrow failure and pulmonary fibrosis (Vulliamy et al., 2001). Other studies also found abnormal telomere shortening in about 25% of random IPF cases and about 12% of familial IPF cases (Petrovski et al., 2017). In addition, signs of telomere shortening have also been found in peripheral blood and lung of non-genetic individuals with Idiopathic interstitial pneumonia (IIP) (Alder et al., 2008). Some IPF or fibrotic hypersensitivity pneumonitis patients with peripheral blood short telomere length have been linked to worse survival (Stock and Renzoni, 2021).

Telomerase in human cells, specific some tumour cells, can bind new pieces of telomere to the end of DNA and achieve infinite cell proliferation (Shay, 2016). It has been proved that shorter telomere length is linked with a higher risk of lung cancer. The telomere length shortening has the greatest impact on the risk of SCLC when classified by lung cancer tissue subtype (Jang et al., 2008). But a study found that individuals with longer telomere length have an increased risk of lung cancer (Doherty et al., 2018). It might be interpreted that the shortening of telomere length increases the genetic instability of cells, so it is prone to genetic mutations and other chromosome abnormalities, which increases the risk of lung cancer. Longer telomere lengths allow cells to live longer and get a chance of increased genetic mutation during frequent cell division. Telomere length is an independent prognostic factor of early-stage NSCLC (Jeon et al., 2014). As an important tumour driver gene, the epidermal growth factor receptor (EGFR) is involve in telomerase activity and enhances TERT transcription (Chen et al., 2017; Daniel et al., 2012; Steelman et al., 2011).

## 6 Chromatin remodeler

The chromatin remodelling complex could intake energy from ATP to reconstruct chromatin. Thus, such remodelers can regulate a series of cellular processes including chromatin accessibility, gene transcription, DNA replication and DNA damage responses (Reyes et al., 2021; Tyagi et al., 2016). Eukaryotic cells contain four families of chromatin remodelers, including switch/sucrose nonfermentable (SWI/SNF), imitation switch (ISWI), chromodomain helicase DNA-binding (CHD), and Inositol requiring 80 (INO80) (Flaus et al., 2006).

SMARCA family also played a role in chromatin remodelling, especially in double-strand damage repair. BRM gene, located on chromosome 9P24.3, is one of two mutually exclusive catalytic subunits in the SWI/SNF chromatin-remodelling complex. Inactivated BRM mutations are rarely detected in cancers, however, cell lines and primary cancers have been found to show little or no BRM protein (Wilson et al., 2014). BRG1 gene is located on chromosome 19P13.2 and encodes BRG1 protein. Another catalytic subunit is BRM, which is encoded by the BRM gene (Chetty and Serra, 2020). BRG1gene is mutated in up to 10%–35% of NSCLC (Kadoch et al., 2013). Concomitant loss of BRG1 and BRM was seen in 10% of non-small cell lung cancers (Reisman et al., 2003). Analysis of a total of 316 lung cancers found complete loss of BRG1 in 5.5% of lung adenocarcinomas and 5.2% of squamous cells, and complete loss of BRM in 6.4% of adenocarcinomas and only 1.7% of squamous cells (Herpel et al., 2017).

Genomic sequencing studies have indicated that mutations in multiple subunits of the SWI/SNF chromatin-remodelling complex, such as BRG1 or BRM as the catalytic subunit, frequently occurred in numerous solid tumours, including lung cancers. Low BRG1 expression levels in primary human NSCLC correlated with overexpression of the NRF2-target gene (Song et al., 2020). Depletion or inhibition of BRG1 downregulated cyclin B1 (CCNB1) and latent TGF-β -binding protein 2 (LTBP2) in lung cancer cells. BRG1 can recruit histone H3K9 demethylase KDM3A, which could remove dimethyl H3K9 from the target gene promoter and activate target genes (Wilson et al., 2014). BRG1 and another subunit of, the SWI/SNF family, ZEB1, both synergistically inhibit the expression of E-cadherin, thereby promoting EMT in tumour cells (Sanchez-Tillo et al., 2010).

ARID1A can bind DNA in a non-sequence-specific manner by alternating nucleosome strength and participates in DNA repair and stabilization processes (Sun et al., 2021; Wang X et al., 2004). Alterations in ARID1A may be diverse and have been observed in many cancers, including lung cancer (Naito et al., 2009; Huang et al., 2015). The downregulation of ARID1A protein expression in NSCLC is correlated with the TNM stage and nodal status. Moreover, the results showed that ARID1A depletion could promote lung cancer cell proliferation and decrease sensitivity to the conventional chemotherapies, possibly by Akt-mediated cyclin D1 and Bcl-2 regulation (Zhang et al., 2014).

The role of chromatin remodelling complexes in organ fibrosis has not been well-studied. Chromatin remodelling factors play an important role in cardiac and renal fibrosis (Naito et al., 2009; Scavello et al., 2021), but their role in pulmonary fibrosis is limited. Deletion of SMARCA4 impairs alveolar epithelial type II cell proliferation and aggravates pulmonary fibrosis in mice (Peng D et al., 2017).

## 7 Conclusion

Epigenetic alternations are closely related to lung diseases and play an important role in their pathogenesis, including DNA methylation, histone modification, lncRNA, ageing and telomere, and chromatin remodelling. For these high case-fatality rates of this dreadful disease, diagnostic and therapeutic approaches targeting epigenetic modifications that could help to improve prognosis, but further researches and clinical application need to carry out. Lung cancer is clinically ineffective, chemotherapy, radiotherapy, targeted therapy, and immunotherapy. Although there is no effective clinical anti-tumour therapy in epigenetic fields, people are actively researching and exploring targeted therapy drugs from different perspectives, such as telomere structure analogues and telomerase inhibitors (Duchler, 2012; Liu et al., 2017; Rankin et al., 2008), looking forward to breakthroughs in lung cancer research.
